# Non-medical use of methylphenidate among medical students of the University of the Free State

**DOI:** 10.4102/sajpsychiatry.v23.1006

**Published:** 2017-01-20

**Authors:** Roshini Jain, Ch Chiech Chang, Mpho Koto, Alden Geldenhuys, Richard Nichol, Gina Joubert

**Affiliations:** 1 School of Medicine, University of the Free State, South Africa; 2 Department of Psychiatry (G66), University of the Free State, South Africa; 3 Department of Biostatistics (G31), University of the Free State, South Africa

## Abstract

**Background:**

Faced with demanding training programmes, medical students may be more prone to use methylphenidate for non-medical purposes in order to improve concentration, alertness and academic performance.

**Aim:**

The study aimed to investigate the prevalence of the non-medical use of methylphenidate and knowledge of this drug among undergraduate medical students of the University of the Free State.

**Methods:**

This was a cross-sectional study. A self-administered, anonymous questionnaire was distributed during lectures to all students in the five year groups of the undergraduate medical programme.

**Results:**

Of the 643 undergraduate medical students, 541 completed the questionnaire (response rate: 84.1%). Approximately 11.0% of surveyed students were using methylphenidate at the time of the study, of which the majority (67.9%) used it for academic purposes and 70.6% received it from a medical health professional. Less than a third of users had been diagnosed with Attention-Deficit/Hyperactivity Disorder. Methylphenidate users’ median knowledge was greater than non-users, and methylphenidate knowledge increased from first-year and second-year students to third-year to fifth-year students. Median knowledge scores per year group ranged from 52.0% to 60.0%.

**Conclusion:**

Methylphenidate is mainly used for non-medical purposes by medical students. Students generally have a low level of knowledge on methylphenidate. Specific information on methylphenidate should be included in lectures on stress management and study methods during the course of the medical curriculum.

## Introduction

Methylphenidate is used mainly for the treatment of Attention-Deficit/Hyperactivity Disorder (ADHD) and narcolepsy.^1^ To date, it is the only stimulant Schedule-6 drug available for these purposes in South Africa. Numerous studies have, however, found that healthy high school and university students use methylphenidate to improve concentration, alertness and academic performance, or for recreational purposes.^2,3,4,5^ The term ‘non-medical use’ of prescription drugs is defined by the United Nations Office on Drugs and Crime as the taking of prescription drugs, whether obtained by prescription or otherwise, other than in the manner or for the reasons or time period prescribed, or by a person for whom the drug was not prescribed.^6^ In contrast, off-label use in the United States (US) implies that the regulations of the Food and Drug Administration (FDA) permit physicians to prescribe approved medications for other than their intended indications, and misuse refers to the wrong, improper use or misapplication.^7,8^

In 2009, Bogle and Smith^9^ reported that the self-reported rates among various studies among US college students ranged from 1.5% to 31.0%. In 2015, Arria,^10^ using her personal findings as a researcher in the College Life Study, and quoting the data of the American College Health Association, considered the incidence of stimulant use in college students to be 10.0%. In the US, stimulants may be used in a compensatory fashion to increase students’ sub-standard academic performance. Students may use other illicit drugs, drink heavily, avoid attending classes and not utilise the necessary time for studying, and then turn to prescription stimulants as a way to compensate for their behaviour.^10^ A New Zealand study^11^ of third-year university students in 2015 reported a rate of 6.6% for the use of cognitive enhancers, including methylphenidate. More recently, a South African study^12^ reported that 17.2% of undergraduate students at one university had used methylphenidate with only 2.9% being diagnosed with ADHD.

Evidence suggests that medical students may be prone to alcohol and drug use.^13^ Faced with demanding training programmes, these students have fears and anxiety about failing, and may feel the pressure to perform. Emanuel et al.^13^ in 2013 reported that 11.0% of medical students from four medical schools in the Chicago area indicated use of cognitive enhancers of which 41.0% used methylphenidate. In 2015, Cohen et al.^14^ reported that among Israeli medical students, 13.5% indicated methylphenidate use during the past year. Approximately 10.0% of students reported ever being diagnosed with ADHD. A recent study done on second-year and fourth-year medical students at one South African university found 13.1% (33/251) reporting substance use for non-medical purposes during the preceding year, with only 2.0% reporting an ADHD diagnosis.^15^

Students using methylphenidate can experience numerous side-effects, such as hallucinations, anxiety, dry mouth and visual disturbances.^2^ Withdrawal symptoms can include fatigue, disturbed sleeping patterns and depression, while incorrect doses can result in cardiovascular failure or lethal seizures.^1^

This study investigated the prevalence of non-medical use and the extent of their knowledge of methylphenidate among undergraduate medical students of the University of the Free State (UFS). Additional objectives included comparing the prevalence of methylphenidate misuse among the different year groups and determining other stimulants most commonly used.

## Research methods and design

### Study design and population

This was a cross-sectional study. The study population consisted of first-year to fifth-year undergraduate medical students at the School of Medicine, UFS, during 2013. The sample size was 643 students. Students who were not present on the day of the survey were excluded from the study. There was no intervention.

### Data collection

A self-administered and anonymous questionnaire was designed by the researchers based on clinical experience of the study leader and information from the literature to test the participants’ knowledge regarding symptoms, positive effects and drug scheduling of methylphenidate. Data on demographic information and usage and knowledge of methylphenidate were collected. The questionnaire was available in English and Afrikaans. Questionnaires were distributed to each year group during lectures. Students were asked to put their completed questionnaires in a box provided after immediate completion.

### Pilot study

A pilot study was conducted to test the quality of both the English and Afrikaans questionnaires. Ten first-year and 10 second-year physiotherapy students were randomly selected to answer both the questionnaires. After the pilot study, some of the questions were rephrased for clarity, and grammar errors in the Afrikaans questionnaire were corrected. Additional options were provided to ensure that all possible choices were covered. Data from the pilot study were excluded from the main dataset.

### Data analysis

The data were analysed by the Department of Biostatistics, Faculty of Health Sciences, UFS, using SAS Version 9.1. Categorical information was summarised by frequencies and percentages, and numerical variables by means, standard deviations or percentiles (based on the distribution of data). Subgroups were compared using chi-squared or Fisher’s exact tests (categorical variables, depending on size of cells) and Kruskal–Wallis tests (skew numerical variables).

The questionnaire contained a screening question for each section, for example, ‘Have you ever heard of methylphenidate (e.g. Ritalin^®^)?’, or ‘Do you use methylphenidates, for example, Ritalin^®^?’ If a participant answered ‘No’, but from subsequent questions it was clear that the answer could be ‘Yes’, a separate category was created: ‘No, but continued’. A total knowledge score was calculated based on the answers to five knowledge questions. One of the knowledge questions contained a list of symptoms from which the correct ones had to be identified. This question was scored as a proportion out of one based on the number of correct choices. The answers to the other four questions regarding knowledge were scored as either correct or incorrect. The overall total knowledge score was thus represented as a percentage correct out of five.

### Ethical considerations

The protocol was approved by the Ethics Committee of the Faculty of Health Sciences, UFS. Permission was obtained from the Vice-Rector Academic, Dean of the Faculty of Health Sciences, and Head of the School of Medicine, UFS.

Participation in the study was voluntary and students could withdraw from the study at any point. An information leaflet was provided in Afrikaans and English. To protect participants, questionnaires were anonymous. Age and marital status at the time of study were not asked in order to ensure anonymity. Owing to the anonymous nature of the questionnaire, completion of the questionnaire was considered as consent for participation.

## Results

Of the 643 undergraduate medical students, 541 completed the questionnaire (response rate: 84.1%): 143 first-year (response rate: 91.7%), 114 second-year (response rate: 88.4%), 92 third-year (response rate: 84.4%), 101 fourth-year (response rate: 80.8%) and 91 fifth-year students (response rate: 73.4%). More than half (*n* = 294; 54.3%) of the participants were female. This was the case for all year groups except the fifth-year students of whom 47.3% were female.

Most (*n* = 474; 87.6%) of the students had ever heard about methylphenidate, 10.5% (*n* = 57) had not heard and 1.9% (*n* = 10) said that they had not heard about methylphenidate but continued to complete the section on methylphenidate use (Table 1). The highest percentage (18.5%) of students using methylphenidate was in the third-year group. The main sources of information on methylphenidate were friends (58.1%) and family (40.5%), followed by mass media (26.7%) (more than one option could be chosen). Students also heard about methylphenidate from medical books (22.5%), a medical doctor (19.8%), the Internet (17.4%), a scientific journal (3.5%) and other sources (4.3%).

**TABLE 1:** Percentage of students across year groups and overall who had heard about methylphenidate and had used this stimulant.

Variable	First-year	Second-year	Third-year	Fourth-year	Fifth-year	Total
	*n* (%)	*n* (%)	*n* (%)	*n* (%)	*n* (%)	*n* (%)
Had heard about methylphenidate	*n* = 143	*n* = 114	*n* = 92	*n* = 101	*n* = 91	*n* = 541
Yes	123 (86.0)	97 (85.1)	80 (87.0)	91 (90.1)	83 (91.2)	474 (87.6)
No, but continued†	0 (0)	4 (3.5)	1 (1.1)	4 (4.0)	1 (1.1)	10 (1.9)
No	20 (14.0)	13 (11.4)	11 (12.0)	6 (5.9)	7 (7.7)	57 (10.5)
Used methylphenidate	*n* =123	*n* =101	*n* =81	*n* =94	*n* =84	*n* =483
Yes	13 (10.6)	6 (5.9)	15 (18.5)	10 (10.6)	4 (4.8)	48 (9.9)
No, but continued?	0 (0)	1 (1.0)	0 (0)	1 (1.1)	3 (3.6)	5 (1.0)
No	110 (89.4)	94 (93.1)	66 (81.5)	83 (88.3)	77 (91.7)	430 (89.0)

† Indicated that they had not heard about methylphenidate but continued to complete the questions related to methylphenidate use.

Overall, 53 students were considered to have used methylphenidate of whom 30.2% had been diagnosed with ADHD, whereas 70.6% obtained methylphenidate via a prescription from a medical doctor. The majority (67.9%) used methylphenidate to improve academic results (more than one option could be chosen). Just over half (54.7%) of the 53 students used methylphenidate during high academic stress periods, and 24.5% used it daily. Few students (9.4%) used methylphenidate in combination with alcohol. Most students (62.7%) started using methylphenidate between ages 19 and 22 years. Since the number of users per year group vary between seven (second- and fifth-year students) and 15 (third-year students), the above results are not reported per year group.

Overall, the majority of students knew that methylphenidate is illegal if obtained without prescription (86.6%), positive effects last only for a few hours (66.3%) and that users can become dependent (65.4%) (Table 2). Fifth-year students had the highest correct responses for the aspect of illegality and effects lasting only a few hours, namely 95.2 and 77.1%, respectively. For the question regarding dependence, less than 60.0% of the fifth-year and first-year students answered correctly compared with more than 70.0% of the other year groups. Few students knew that methylphenidate is a Schedule-6 medication (23.7%). Fifth-year students had the highest percentage of correct answers for the schedule of medication, namely 36.9%, but an equal percentage identified it as a Schedule-5 medication.

**TABLE 2:** Knowledge per year of study.

Variable	First-year *N* = 123	Second-year *N* = 101	Third-year *N* = 81	Fourth-year *N* = 95	Fifth-year *N* = 84	Total *N* = 484	*p*
	*n* (%)	*n* (%)	*n* (%)	*n* (%)	*n* (%)	*n* (%)	
Symptoms of the side-effects of methylphenidate use (multiple options could be selected)†							
Sleepiness	18 (14.6)	18 (17.8)	6 (7.1)	11 (11.6)	17 (20.2)	70 (14.5)	0.1314
Nervousness	21 (17.1)	35 (34.7)	25 (30.9)	49 (51.6)	31 (36.9)	161 (33.3)	< 0.0001
Dizziness	8 (6.5)	3 (3.0)	8 (9.9)	6 (6.3)	10 (11.9)	35 (7.2)	0.1630
Cardiac arrhythmia	22 (17.9)	28 (27.7)	24 (29.6)	42 (44.2)	27 (32.1)	143 (29.6)	0.0011
Memory loss‡	2 (1.6)	6 (5.9)	6 (7.4)	10 (10.5)	5 (6.0)	29 (6.0)	0.0946
Erectile dysfunction‡	3 (2.4)	3 (3.0)	5 (6.2)	15 (15.8)	2 (2.4)	28 (5.8)	0.0001
Palpitations	26 (21.1)	28 (27.7)	32 (39.5)	55 (57.9)	39 (46.4)	180 (37.2)	< 0.0001
I don’t know	52 (42.3)	42 (41.6)	29 (35.8)	20 (21.1)	20 (23.8)	163 (33.7)	0.0015
None	9 (7.3)	5 (5.0)	4 (4.9)	8 (8.4)	8 (9.5)	34 (7.0)	0.6793
Schedule category of methylphenidate (*n* = 2 missing)							< 0.0001
Schedule 6	23 (18.9)	21 (20.8)	19 (23.8)	20 (21.1)	31 (36.9)	114 (23.7)	
Other schedule	21 (17.2)	24 (23.8)	24 (30.0)	39 (41.1)	39 (46.4)	147 (30.5)	
I don’t know	78 (63.9)	56 (55.5)	37 (46.3)	36 (37.9)	14 (16.7)	221 (45.9)	
Methylphenidate is an illegal drug if obtained without prescription							0.1510
True	101 (82.1)	83 (82.2)	71 (87.7)	84 (88.4)	80 (95.2)	419 (86.6)	
False	5 (4.1)	2 (2.0)	2 (2.5)	3 (3.2)	2 (2.4)	14 (2.9)	
I don’t know	17 (13.8)	16 (15.8)	8 (9.9)	8 (8.4)	2 (2.4)	51 (10.5)	
Positive effects of methylphenidate lasts only for several hours (*n* = 1 missing)							0.0040
True	81 (65.9)	56 (55.5)	51 (63.0)	68 (71.6)	64 (77.1)	320 (66.3)	
False	9 (7.3)	8 (7.9)	10 (12.4)	14 (14.7)	7 (8.4)	48 (9.9)	
I don’t know	33 (26.8)	37 (36.6)	20 (24.7)	13 (13.7)	12 (14.5)	115 (23.8)	
Methylphenidate has no characteristics of addictive drugs, and users do not become dependent (*n* = 4 missing)							< 0.0001
True	15 (12.4)	3 (3.0)	6 (7.5)	11 (11.7)	24 (28.6)	59 (12.3)	
False	69 (57.0)	71 (70.3)	56 (70.0)	68 (72.3)	50 (59.5)	314 (65.4)	
I don’t know	37 (30.6)	27 (26.7)	18 (22.5)	15 (16.0)	10 (11.9)	107 (22.3)	

† The results indicate *n*; % who chose the symptom;

‡ Incorrect symptoms.

Overall, 7.0% of students indicated that methylphenidate use had no side-effects, and 33.7% indicated that they did not know what the symptoms of side-effects were. Less than a quarter of the fourth-year and fifth-year students indicated that they did not know either. The symptoms most commonly identified were palpitation (37.2%), nervousness (33.3%) and cardiac arrhythmia (29.6%). First-year students had the lowest percentages of approximately 20.0%. Sleepiness and dizziness were only identified by 14.5% and 7.2%, respectively.

According to Table 3, the median knowledge of students who used methylphenidate was higher compared with students who did not use methylphenidate. This difference was significant for first-year and third-year students (*p* < 0.05). The median marks increased significantly from the first-year and second-year students to the third-year to fifth-year students (*p* = 0.0008).

**TABLE 3:** Median knowledge (%) of students who use and those who do not use methylphenidate.

Year of study	Median knowledge (%)		
	Students using methylphenidate	Students not using methylphenidate	Total
First-year	64	48	52 (IQR 40; 64)
Second-year	64	50	52 (IQR 40; 64)
Third-year	72	48	60 (IQR 40; 68)
Fourth-year	68	60	60 (IQR 44; 68)
Fifth-year	72	60	60 (IQR 44; 72)

IQR, Interquartile range.

The majority (*n* = 381; 70.8%) of students reported using non-prescribed stimulants, excluding methylphenidate, to help them concentrate on their studies. These included coffee (81.6%); supplements that increased mental vitality and concentration (45.4%); energy drinks (39.1%); and other stimulants such as tea, cigarettes and sweets (9.2%). Methylphenidate users were not more likely than non-users to report using other non-prescribed stimulants overall (26.4% vs. 27.3%), but methylphenidate users were significantly more likely to also use energy drinks (39.6% vs. 26.7%, *p* = 0.049).

## Discussion

The main reason for students using methylphenidate was to improve their academic results.

Other studies found a decrease in use for academic purposes as the years progressed,^2^ but this could not be investigated reliably in our study owing to small numbers of users per year. The percentage users (for whichever reason) showed no clear trend over the year groups, for example, the highest percentage users were third-year students, and the lowest percentage were second-year students.

At our medical school, students receive no training regarding methylphenidate use during the pre-clinical years of study (the first five semesters of the 10-semester programme), whereas students in the clinical years (the last five semesters of the programme) would have exposure to this subject during rotations through Paediatrics and Psychiatry. It is therefore expected that fourth-year and fifth-year students would have better knowledge of methylphenidate use.

In general, those who used methylphenidate tended to have more knowledge on its side-effects. In an Iranian study, the mean knowledge of students using this stimulant was also found to be higher than the mean knowledge of non-users.^5^

### Study limitations

The questionnaires were anonymous and self-administered, and we were therefore dependent on students’ honesty when answering the questionnaires. The questionnaire consisted of six pages with 29 questions (a second section not reported on here dealt with cannabis use and knowledge). The survey was conducted in class before the lecture started, so the students may have been in a rush to finish the questionnaires.

To differentiate between the use and misuse of methylphenidate, participants were asked whether they were diagnosed with ADHD. From this, the assumption was made that participants who were not diagnosed with ADHD were misusing methylphenidate. However, the questionnaire was not structured in such a way to determine whether the participants with ADHD were using methylphenidate as prescribed or misusing it for reasons not related to their ADHD.

## Conclusion

The study demonstrated that students use methylphenidate for non-medical purposes, mainly to improve academic results. Senior students have better knowledge regarding the drug. Students who use methylphenidate have greater knowledge of it than those who do not use it. The most commonly used, legally accepted stimulant is caffeine.

The medical curricula should include methylphenidate misuse when presenting lectures on study methods and how to cope with stress during the first-year. Students should be warned of the wrongful use of methylphenidate and also the effects of caffeine, such as sleep disturbances, considering how important sleep is for memory. Ethics lectures can also deal with aspects regarding the medical prescribing of cognitive enhancers without medical grounds.
